# Phyto-Mediated Versus Conventional Nanomaterials for Environmental Remediation: Surface Chemistry, Removal Mechanisms, Performance, and Sustainability

**DOI:** 10.3390/nano16171118

**Published:** 2026-09-04

**Authors:** Farhah Elfadel Omer, Aliaa Alrashidi, Akeem Omolaja Akinfenwa, Amani M. Alansi, Mohammed S. Alotaibi, Adebayo Adekunle Rasheed, Bader Alharbi, Fatehia S. Alhakami, Idris K. Popoola, Talal F. Qahtan

**Affiliations:** 1 Physics Department, College of Science and Humanities in AI-Kharj, Prince Sattam Bin Abdul Aziz University, Al-Kharj 11942, Saudi Arabia; 2 Department of Physics, College of Science, Qassim University, Buraydah 52571, Saudi Arabia; aliaa.alrashidi@qu.edu.sa; 3 Department of Chemical Science, School of Science, Yaba College of Technology, P.M.B 2011, Yaba, Lagos State, Nigeria; 4 Chemistry Department, College of Science, Taiz University, Taiz 12372, Yemen; mn.alansi2016@gmail.com; 5 Federal University of Technology, P.M.B. 65, Minna, Niger State, Nigeria; 6 Et al. Technologies Limited, Gbagada 100234, Lagos State, Nigeria; popoola.idris.k@gmail.com

**Keywords:** nanomaterials, phyto-mediated synthesis, green nanotechnology, surface chemistry, environmental remediation, water treatment, adsorption, photocatalysis, sustainability

## Abstract

Nanomaterials have emerged as key platforms for environmental remediation owing to their tunable surface chemistry, high specific surface area, and multifunctional physicochemical properties. This review provides a critical comparison between conventional nanomaterials (CNMs) and phyto-mediated nanomaterials (PMNs), with particular emphasis on material design, surface chemistry, pollutant removal mechanisms, environmental performance, and sustainability. CNMs, including metal and metal oxide nanoparticles, carbon-based nanomaterials, and hybrid nanocomposites, offer excellent adsorption, photocatalytic, redox, and antimicrobial performance but remain constrained by concerns regarding toxicity, environmental persistence, and energy-intensive synthesis. PMNs provide a greener alternative by integrating plant-derived surface chemistry with nanomaterial functionality, potentially reducing reliance on hazardous synthesis reagents while modifying interfacial interactions relevant to environmental remediation. The review critically discusses the mechanistic roles of adsorption, surface complexation, photocatalytic degradation, electron-transfer processes, and reactive oxygen species (ROS) generation in pollutant removal. Recent advances in water purification, soil and groundwater remediation, carbon sequestration, and climate-related environmental applications are comprehensively summarized. Finally, current challenges associated with reproducibility, scalability, environmental safety, life-cycle assessment, and regulatory considerations are critically analyzed, together with future perspectives toward the rational design of sustainable nanomaterials for next-generation environmental remediation technologies.

## 1. Introduction

Access to clean water and a healthy environment remains one of the most pressing global challenges. Rapid industrialization, urban expansion, agricultural activities, and population growth have significantly increased the release of contaminants into aquatic and terrestrial systems. Pollutants such as heavy metals, dyes, pesticides, and pathogenic microorganisms pose serious risks to human health and environmental sustainability [1,2]. Conventional water and wastewater treatment technologies, including coagulation, flocculation, biological treatment, adsorption, and chemical oxidation, have contributed substantially to pollution control. However, many of these methods suffer from limitations related to efficiency, selectivity, cost, and secondary pollution, particularly when dealing with emerging contaminants [3,4].

**Figure 1:**
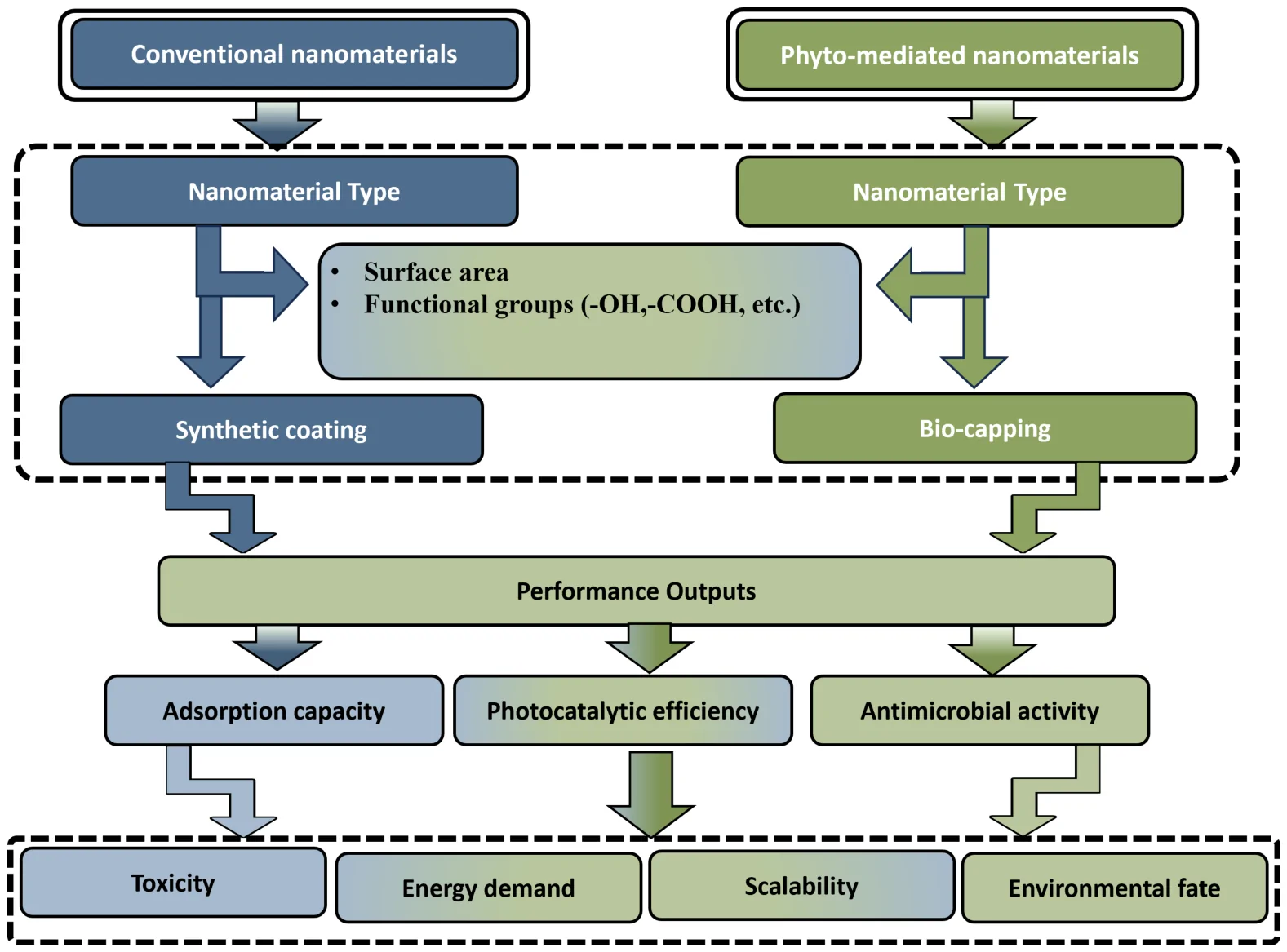
Structure–performance–sustainability framework comparing CNMs and PMNs, illustrating how differences in surface modification influence remediation performance and key sustainability considerations.

Recent advances in nanochemistry offer promising alternatives by exploiting size-dependent physicochemical properties at the nanoscale. Materials with dimensions between 1 and 100 nm exhibit high surface-to-volume ratios, tunable surface chemistry, and enhanced catalytic activity, enabling efficient pollutant removal through adsorption, photocatalysis, redox transformation, and antimicrobial processes [5,6]. Despite these advantages, concerns regarding the environmental impact, toxicity, and sustainability of conventional nanomaterials (CNMs) have driven increasing interest in greener approaches. In this context, phyto-mediated nanomaterials (PMNs) have emerged as a promising alternative by employing plant-derived components during nanomaterial synthesis. The resulting differences in synthesis route and surface chemistry can influence material functionality, environmental interactions, and remediation performance. To provide a clear conceptual understanding of the relationship between material design, performance, and environmental sustainability, a comparative framework is presented in Figure 1 The framework illustrates how differences in nanomaterial type and surface modification, particularly synthetic coating in CNMs and bio-capping in PMNs, influence surface characteristics and subsequently affect adsorption, photocatalytic, and antimicrobial performance. These performance outcomes are further linked to key sustainability considerations, including toxicity, energy demand, scalability, and environmental fate.

In phyto-mediated synthesis, plant extracts function as natural reducing, stabilizing, and capping agents, enabling the formation of nanomaterials under mild and eco-friendly conditions [7,8]. This review therefore critically examines how differences between conventional and phyto-mediated synthesis translate into variations in surface chemistry, remediation mechanisms, environmental performance, and sustainability. Accordingly, the review provides a comparative analysis of CNMs and PMNs, covering their fundamental principles and classifications, applications in water, soil, groundwater, and climate-related environmental systems, and the mechanisms governing pollutant removal. Particular attention is given to adsorption and surface complexation, photocatalytic and redox processes, antimicrobial mechanisms, and interactions with environmental matrices. Finally, challenges related to reproducibility, scalability, long-term environmental safety, life-cycle considerations, and regulatory implementation are critically assessed to identify priorities for the future development and responsible deployment of sustainable nanomaterials.

## 2. Fundamentals of Nanochemistry in Environmental Applications

Nanochemistry explains how surface atoms of nanostructured material account for the surface energy, altered electronic structure, and enhanced chemical reactivity [9]. These size-dependent and tunable electronic characteristics have continuously been explored in nanotechnology research for water treatment and environmental remediation. Although nanochemistry and nanotechnology are often used interchangeably, nanotechnology is a broad field that encompasses the design, fabrication, and utilization of nanoscale materials and devices across multiple disciplines. In contrast, nanochemistry as discussed applies chemical principles and focuses on the synthesis, composition, surface modification, and reactivity of materials at the nanoscale [10]. Thus, nanochemistry and nanotechnology are complementary concept of particular relevance in environmental applications involving removal of pollutants and chemical interactions at nano surface-interfaces between analytes and the removing agent.

Nanochemistry in environmental application relates to the emergence of size-dependent physicochemical properties of materials used for environmental remediation. As particle dimensions decrease, changes in surface-to-volume ratio, quantum confinement, and surface coordination lead to modified optical, electronic, catalytic, and adsorption behaviors. These effects result in higher reactivity and improved interaction with contaminants and efficient removal of heavy metals, organic pollutants, and microbial species from water matrices [11]. Such size-dependent behavior, together with surface functionalization, determines the surface chemistry of nanomaterials through functional groups such as hydroxyl, carboxyl, amine, and thiol moieties on nanoparticle surfaces. Through deliberate surface modification of plant (stabilizing agents) during synthesis, nanomaterials can be fabricated to exhibit enhanced selectivity, hydrophilicity, and binding affinity toward pollutants and stability in complex aqueous environments necessary for adsorption and catalysis-based treatment processes [12,13]. Furthermore, nanointerfaces facilitate processes such as electrostatic attraction, surface complexation, redox reactions, and photocatalytic degradation. The high density of reactive sites at these interfaces enables rapid and efficient pollutant transformation, making chemical enabled nanomaterials suitable for water purification and environmental remediation applications. With the current emphasis placed on green and sustainable nanochemistry to address concerns related to environmental safety and long-term impacts. Green nanochemistry approach used in the synthesis and application of PMNs provides alternative routes to conventional method of water treatment an environmental remediation. This strategy applies benign solvents, low-cost synthesis routes, and environmentally compatible surface modifiers is increasingly recognized as essential for the responsible deployment of nano chemical solutions in environmental systems [14,15].

## 3. Nanomaterials for Water Treatment and Environmental Remediation

**Figure 2:**
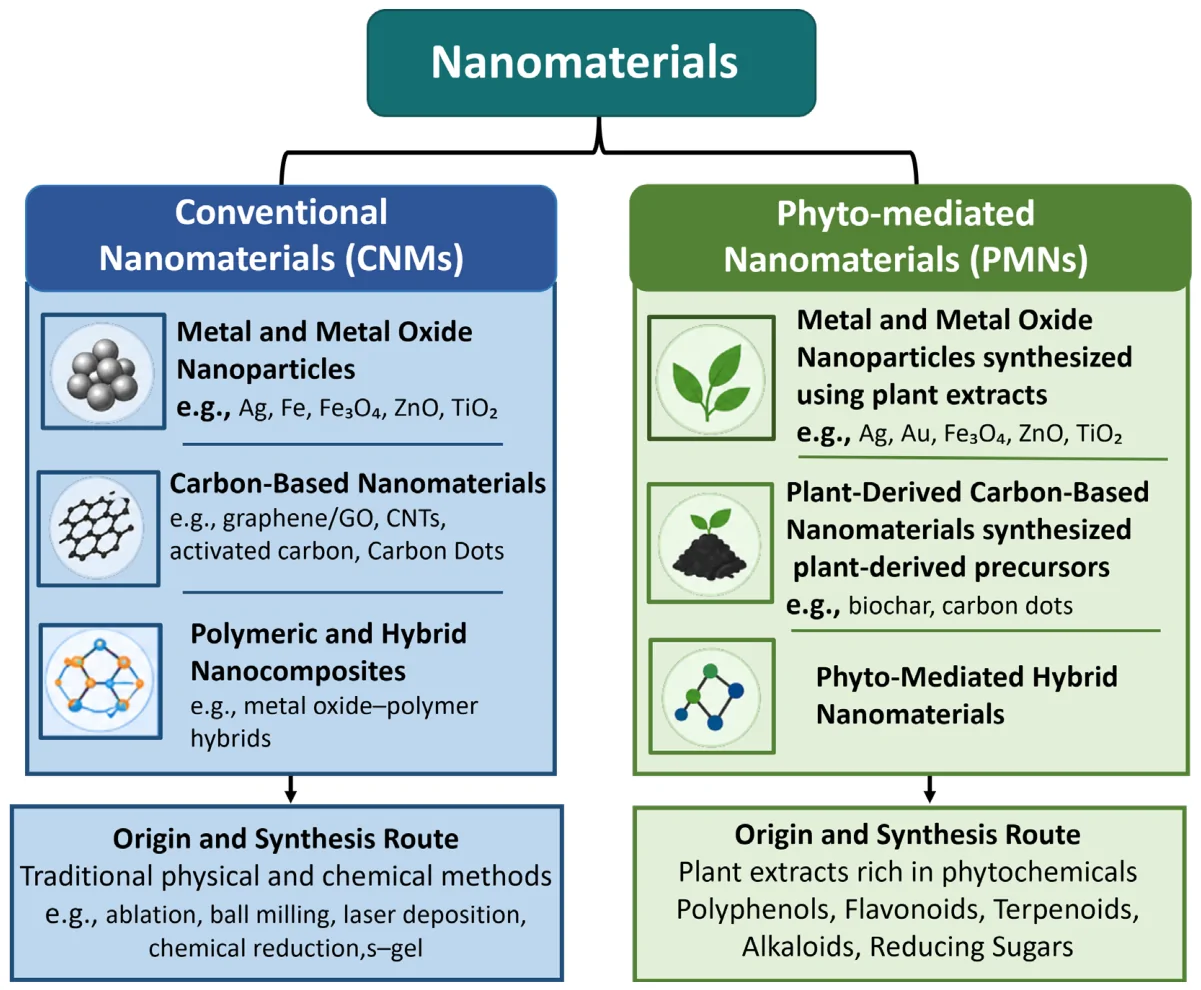
Classification of conventional nanomaterials (CNMs) and phyto-mediated nanomaterials (PMNs) based on their origin and synthesis routes for environmental remediation.

As illustrated in Figure 2, nanomaterials employed in water treatment and environmental remediation are classified as CNMs and PMNs, based on their origin and synthesis routes [16]. Conventional also known as traditional nanomaterials such as ZnO, TiO_2_, ZrO_2_ represent the earliest and most extensively studied category and include metal and metal oxide nanoparticles, carbon-based nanomaterials, and polymeric or hybrid nanocomposites. These materials have demonstrated significant potential in the removal of a wide range of contaminants due to their tunable physicochemical properties and diverse modes of action [17].

### 3.1. Conventional Nanomaterials

Conventional synthesis of nanomaterials applies physical (ablation, ball milling, laser deposition etc.) and chemical reduction procedures to synthesize metal and metal oxide nanoparticles and materials. These include zero-valent metals and bimetallic nanoparticles, carbon-based nanomaterials, polymeric and hybrid nanocomposites. Metal and metal oxide nanoparticles have been widely investigated for their enhanced adsorption and catalytic properties in water treatment [18,19]. Recent reviews of carbon-based nanomaterials, such as graphene and carbon nanotubes (CNTs), highlight their high surface areas and functional group chemistry for pollutant removal. Polymeric and hybrid nanocomposites provide further multi-functionality and improved stability in remediation systems [20]. The application of CNMs in environmental remediation is further discussed.

#### 3.1.1. Metal and Metal Oxide Nanoparticles

Metal and metal oxide nanoparticles, particularly nanoscale zero-valent iron (nZVI), remain among the most widely studied materials for environmental remediation, acting as both adsorbents and photocatalysts for water treatment. They exhibit high reducing potential, enabling electron donation to contaminants and facilitating reductive transformation reactions. For example, Cr(VI) is reduced to less toxic Cr(III), while chlorinated hydrocarbons undergo sequential dechlorination.

Other commonly studied materials include silver, iron, zinc, titanium, and their corresponding oxides such as Fe_3_O_4_, ZnO, and TiO_2_ [21,22]. These nanomaterials demonstrate high surface reactivity, enabling efficient pollutant removal through adsorption, catalytic transformation, redox reactions, and antimicrobial activity. Iron-based nanoparticles, particularly zero-valent iron and iron oxides, have been applied for the removal of heavy metals and chlorinated organic compounds due to their redox activity and magnetic recoverability [23]. Metal oxide nanoparticles such as TiO_2_ and ZnO are widely investigated for the photocatalytic degradation of organic pollutants, where light-induced electron–hole pairs drive oxidative reactions that mineralize contaminants. For instance, Boruah et al. [21] reported the synthesis of magnetic iron oxide nanoparticles containing 7.6 wt.% TiO_2_ on reduced graphene oxide nanosheets, demonstrating enhanced photocatalytic efficiency in the colorimetric detection and removal of persistent atrazine pesticides from polluted water. These dual activities were attributed to the synergistic interaction of Fe_2_O_3_ and TiO_2_ on reduced graphene oxide under irradiation from sunlight [21]. In addition, noble metal nanoparticles, particularly silver nanoparticles (AgNPs), have been widely explored for environmental remediation and microbial disinfection. Their effectiveness is attributed to localized surface plasmon resonance (LSPR) and high surface-area-to-volume ratio, which enable interaction with microbial cells through membrane disruption, reactive oxygen species (ROS) generation, and DNA damage. AgNPs have also been applied for the degradation of organic pollutants such as dyes and pesticides, as well as for the removal of heavy metals including arsenic, lead, and mercury through adsorption and surface-mediated precipitation processes. Despite their effectiveness, concerns regarding the potential toxicity and biodistribution of metal and metal oxide nanoparticles from the synthesis method remain. Hence, several ongoing research toward surface modification, stabilization, and immobilization strategies to improve their environmental safety and practical applications [24,25].

#### 3.1.2. Carbon-Based Nanomaterials

Carbon-based nanomaterials (CBNs) are among the most versatile materials in environmental remediation, consisting of carbon frameworks such as graphene/graphene oxide, carbon nanotubes (CNTs), activated carbon nanostructures, and carbon dots [26]. They are characterized by high surface area, electrical conductivity, chemical stability, and tunable surface chemistry that enhance their adsorption capacity for a wide range of pollutants. The remediation performance of CBNs is primarily attributed to their ability to facilitate electron transfer processes, adsorption, and catalytic reactions. However, pure CBNs typically exhibit limited intrinsic photocatalytic activity, and their effectiveness in photocatalysis often relies on coupling with semiconductor materials. When combined with semiconductors such as TiO_2_, CBNs can act as electron acceptors or charge mediators, improving charge separation and reducing electron–hole recombination. This synergistic interaction enhances the degradation of dyes, pesticides, and organic contaminants through redox reactions [27,28].

Graphene/graphene oxide and CNTs have been widely applied for the removal of heavy metals, dyes, pharmaceuticals, and organic pollutants via π–π interactions, electrostatic attraction, and surface complexation mechanisms. Their structural features, particularly the high aspect ratio and tunable pore structure, make CNTs attractive for membrane-based filtration systems, where selective transport of water molecules is achieved while retaining larger contaminants. Graphene oxide, enriched with oxygen-containing functional groups, exhibits improved hydrophilicity and strong affinity toward pollutants [29,30]. For example, Jain et al. [31] demonstrated that ZnO-decorated graphene oxide forms an efficient and stable photocatalyst for Fenton-based water treatment processes. Similarly, Shams et al. [32] reported enhanced stability of reduced graphene oxide under natural environmental conditions, although photodegradation effects may still influence long-term performance [31,32]. Carbon dots (CDs) represent a distinct subclass of CBNs, typically below 10 nm in size, and are valued for their photoluminescent properties and surface functionality. In addition to remediation, CDs enable pollutant sensing through fluorescence quenching mechanisms. As reported by Ajith et al., CDs can function as dual-purpose materials for both detection and degradation of contaminants, supporting a “detect-and-treat” strategy [33,34]. Despite their advantages, several challenges limit the large-scale application of CBNs, including aggregation in aqueous systems, difficulties in recovery and reuse, and potential environmental and health risks associated with nanocarbon release. Furthermore, the high production cost and energy-intensive synthesis routes of some CBNs, particularly graphene and CNTs, remain significant barriers to practical deployment [35]. These limitations highlight the need for improved functionalization, cost-effective synthesis, and safe disposal strategies.

#### 3.1.3. Polymeric and Hybrid Nanocomposites

Polymeric and hybrid nanocomposites combine the functional advantages of inorganic nanomaterials (e.g., iron oxides) with the flexibility and processability of polymeric matrices such as chitosan. The polymer matrix provides mechanical strength, chemical stability, and structural support, while embedded nanoparticles contribute active sites for contaminant removal. These hybrid systems enable multifunctional behavior, including simultaneous adsorption, catalytic activity, and antimicrobial performance, which is particularly advantageous for treating complex wastewater matrices [36]. The incorporation of biopolymers such as chitosan also represents a shift toward green nanochemistry, as these materials are biodegradable and environmentally compatible. Structurally, hybrid nanocomposites exhibit synergistic architectures, where the polymer matrix stabilizes nanoparticles and reduces aggregation, thereby maintaining surface reactivity and dispersion stability [11]. The reviews by Abdelhamid and Dey et al. indicated the potentials of functional groups like amine (–NH_2_) and hydroxyl (–OH) to act as high-affinity binding sites for divalent heavy metals such as Pb^2+^ and Cd^2+^ in magnetic chitosan–iron oxide nanocomposites, while the iron oxide core enables magnetic separation and recovery from treated water. Beyond the known adsorption systems, advanced fabrication techniques such as electrospinning have been used to produce nanofiber membranes with high porosity and surface area. These structures, along with thin-film nanocomposites (TFNs), are particularly effective in applications such as oil–water separation, desalination, and selective filtration [37,38]. Still, polymeric and hybrid nanocomposites have some limitations including potential polymer degradation under harsh environmental conditions, reduced long-term stability, and possible leaching of embedded nanoparticles. Additionally, scaling up the fabrication of uniform hybrid nanocomposites remains challenging, particularly for membrane-based systems requiring precise structural control. These factors highlight the need for improved material design and standardized fabrication approaches.

### 3.2. Phyto-Mediated Nanomaterials

PMNs are synthesized using plant extracts rich in polyphenols, flavonoids, terpenoids, alkaloids, and reducing sugars, which act as reducing and stabilizing agents during nanoparticle formation [39,40]. These phytochemicals facilitate electron transfer to metal precursors, promoting nanoparticle nucleation and growth while simultaneously forming a bio-capping layer. Functional groups such as hydroxyl and carbonyl moieties bind to nanoparticle surfaces, contributing to colloidal stabilization and reduced aggregation. This biofunctionalization also modifies surface chemistry and pollutant–nanoparticle interactions [41,42]. Compared with chemically synthesized nanoparticles, PMNs may therefore offer advantages associated with milder synthesis conditions, reduced residual chemical toxicity, and naturally derived surface functionality (Table 1). As summarized in Table 1, however, these potential environmental advantages are balanced by limitations in reproducibility, particle-size and morphology control, scalability, and standardization, indicating that the selection between CNMs and PMNs should be application-dependent.

Representative systems include metal and metal oxide nanoparticles such as Ag, Au, Fe_3_O_4_, ZnO, and TiO_2_, as well as carbon-based materials such as biochar and carbon dots derived from plant materials. These systems have been applied to heavy-metal removal, degradation of organic pollutants, and microbial inactivation [43,44,45].

In environmental remediation, PMNs participate in adsorption, catalytic degradation, redox processes, and antimicrobial interactions. Surface-bound phytochemicals can influence electron transfer pathways and ROS generation, thereby affecting remediation performance [46]. PMNs have been investigated for water treatment, soil remediation, and soil organic carbon (SOC) stabilization, where nanoparticle–organic matter interactions may also contribute to carbon sequestration processes [47]. Their plant-derived surface functional groups can further contribute to adsorption, catalytic, and antimicrobial interactions [43].

Despite these potential advantages, several limitations remain. Variability in plant extract composition can affect nanoparticle size, morphology, surface chemistry, and reproducibility, while challenges related to scalability, standardization, and long-term stability may hinder large-scale implementation [48]. Moreover, although PMNs are frequently described as environmentally compatible, their long-term environmental fate, potential ecotoxicity, and interactions with complex ecosystems remain insufficiently understood [49]. These uncertainties highlight the need for improved synthesis control, standardized preparation and characterization protocols, and comprehensive life-cycle and environmental safety assessments before large-scale deployment.

## 4. Applications of Phyto-Mediated Nanomaterials in Environmental Systems

**Figure 3:**
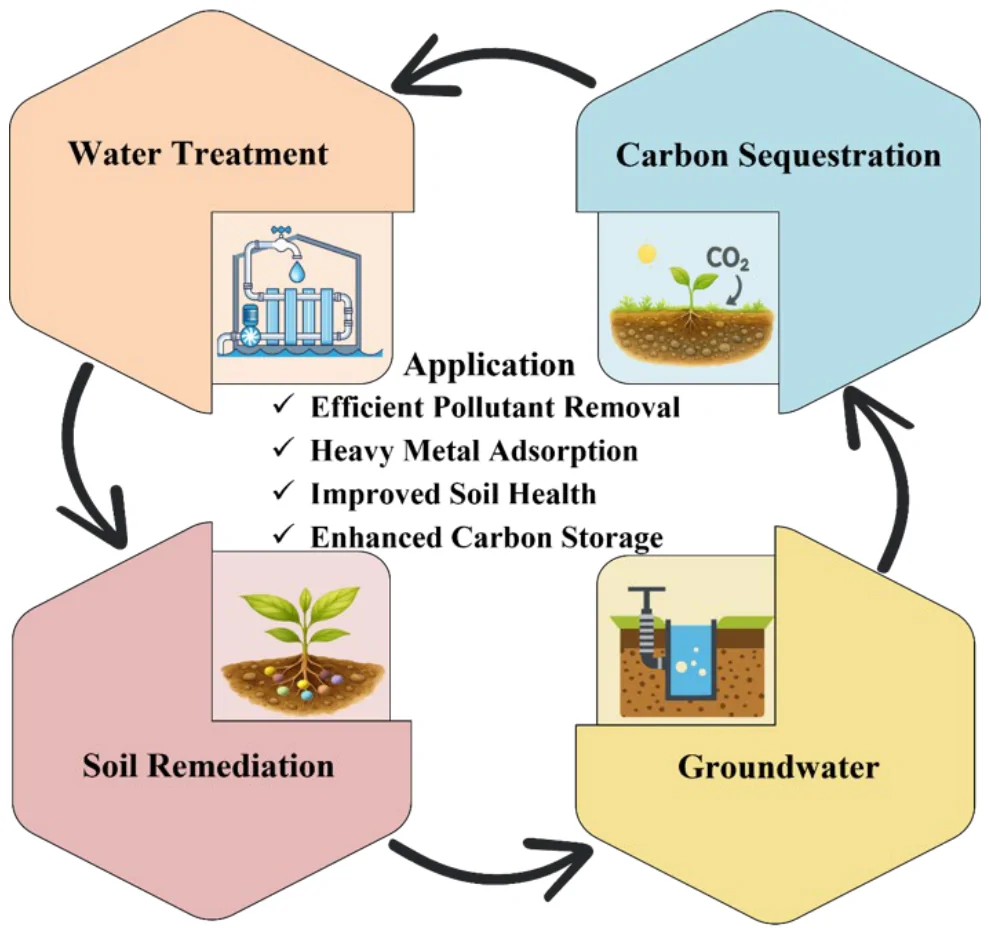
Schematic representation of the key environmental applications of phyto-mediated nanomaterials, highlighting their roles in water and wastewater treatment, soil remediation, groundwater purification, and carbon sequestration.

PMNs have gained increasing attention due to their versatile applications in environmental systems. Their unique combination of surface reactivity, bio-functionalization, and environmentally friendly synthesis enables their use across multiple remediation pathways. These materials have been successfully applied in water and wastewater treatment, soil remediation, groundwater purification, and emerging areas such as carbon sequestration. The main environmental applications of PMNs are summarized in Figure 3, highlighting their multifunctional roles in addressing complex environmental challenges. In the following sections, specific applications related to water treatment, including heavy metal removal, degradation of organic pollutants, and microbial decontamination, are discussed in detail.

### 4.1. Water and Wastewater Treatment

Water sustainability involves practices that ensure the long-term availability of safe and clean water for industrial and ecological needs. However, increasing industrial activities and human–environment interactions have led to significant water contamination by heavy metals, dyes, and organic pollutants, which remain major challenges for conventional treatment methods [50]. PMNs have emerged as a promising branch of phyto-nanotechnology, attracting increasing attention for water and wastewater treatment due to their multifunctional properties, high surface reactivity, and environmentally benign synthesis routes. The incorporation of bioactive phytochemicals onto nanoparticle surfaces enhances interactions with a wide range of contaminants and enables efficient treatment of complex aqueous systems.

Previous studies including Lazim et al. [51] and Navada et al. [52] have reported diverse applications of PMNs in environmental systems, including water purification, soil remediation, and emerging applications in agriculture and healthcare. In this review, particular focus is placed on their application in water and wastewater treatment, including heavy metal removal, degradation of dyes and organic pollutants, and microbial decontamination, alongside the underlying mechanisms responsible for these processes. Despite their promising performance, challenges remain regarding consistency of performance in complex water matrices, potential variability in material properties, and limited large-scale implementation. These limitations highlight the need for further optimization and standardization.

#### 4.1.1. Removal of Heavy Metals

The persistent contamination of water bodies by heavy metals such as lead (Pb), cadmium (Cd), mercury (Hg), and arsenic (As) represents a major environmental challenge. Industrialization and urbanization have increased the release of these toxic elements beyond permissible limits, leading to ecological damage and serious human health risks, including neurodegeneration, respiratory disorders, and kidney dysfunction due to bioaccumulation. Conventional treatment methods for heavy metal removal include coagulation–flocculation, chemical precipitation, ion exchange, adsorption–desorption processes, and membrane filtration. However, these approaches often face limitations related to selectivity, cost, and secondary waste generation [53,54].

With recent advances in green nanochemistry, PMNs have emerged as a promising alternative, exhibiting strong affinity toward heavy metal ions. This enhanced performance is primarily attributed to the presence of surface functional groups (–OH, –CONH–, –COOH, –NH_2_, and phenolic groups) introduced by plant-derived capping agents. These functional groups enable metal ion removal through ion exchange, chelation, and surface complexation mechanisms, facilitating efficient adsorption and separation from aqueous systems [55,56]. As a result, phyto-mediated metal and metal oxide nanoparticles often demonstrate enhanced adsorption capacity and improved selectivity compared to their chemically synthesized counterparts. For example, Emeribe et al. reported synthesis of manganese–magnesium (Mn–Mg) binary oxide nanoparticles using aqueous extracts of *Ficus exasperata* leaves, demonstrating high metal-binding capacity for the removal of Zn, Ni, Fe, Cu, and Cr from contaminated systems through adsorption, oxidation, and co-precipitation mechanisms [56].

Similarly, Pramilaa Kumar and Venkat Kumar reported that Mg-doped iron nanoparticles synthesized from *Hydrocotyle umbellata* leaves exhibited higher adsorption capacity for Ni^2+^ compared with conventional iron oxide and commercially available nanoparticles [57]. Table 2 summarizes representative studies demonstrating the application of PMNs across different environmental media and pollutant classes, including heavy metals and organic contaminants. Collectively, these studies indicate that remediation performance varies with plant source, nanomaterial composition, target pollutant, and treatment medium, making direct comparison across studies dependent on the experimental conditions employed. Despite these promising results, challenges remain, including variability in remediation performance due to differences in plant extract composition, potential competition between co-existing contaminants in real environmental matrices, and limited data on long-term stability and regeneration efficiency. Addressing these limitations is essential for practical large-scale application.

#### 4.1.2. Degradation of Dyes and Organic Pollutants

Mitigating environmental pollution from industrial wastewater contaminated with synthetic dyes and organic pollutants is essential for protecting aquatic and terrestrial ecosystems. Synthetic dyes, pesticides, pharmaceuticals, and other organic contaminants are widely detected in wastewater and are often resistant to conventional treatment methods, including physical and chemical processes of coagulation–flocculation, adsorption, filtration, sedimentation, and flotation, oxidation–reduction, and precipitation [64,65]. These classical methods suffer limitations such as incomplete degradation and the transfer of pollutants from one phase to another rather than complete mineralization. As a result, increasing attention has been directed toward phyto-mediated nanomaterials, particularly those based on metal oxides and carbon systems synthesized using plant extracts. PMNs provide biofunctionalized surfaces with enhanced dispersion and accessibility of active sites, facilitating photocatalytic, catalytic, and redox-driven degradation pathways [39]. Under suitable conditions, these materials generate ROS that can break down complex organic molecules into less toxic intermediate or fully mineralized products. Rashed et al. reported the use of green-synthesized silver nanoparticles derived from *Trigonella hamosa* leaf extracts for the photodegradation of methylene blue and paracetamol in water. Under UV irradiation, degradation efficiencies of 94.49% and 92%, respectively, were achieved, demonstrating the effectiveness of PMNs in treating organic pollutants [58].

Importantly, the photocatalytic potential of phyto-mediated systems extends beyond metallic nanoparticles to semiconductor metal oxides such as ZnO and ZrO_2_. ZnO nanoparticles synthesized using Cosmos caudatus leaf extract achieved 81.5% degradation of methylene blue within 75 min, demonstrating the suitability of plant-mediated ZnO for photocatalytic treatment of organic pollutants [66]. Similarly, ZrO_2_ nanoparticles prepared using plant extracts, including Vitis vinifera, Aloe barbadensis, and Jasminum officinale, exhibited substantial photocatalytic activity. In particular, J. officinale-mediated ZrO_2_ achieved approximately 88% and 90% photodegradation of methylene blue and methyl orange, respectively [67]. These findings demonstrate that green synthesis can produce photoactive ZnO and ZrO_2_ nanomaterials with substantial potential for pollutant degradation.

Despite these promising results, several limitations remain, including dependence on light source intensity, reduced efficiency in real wastewater systems containing mixed contaminants, and potential challenges related to catalyst recovery and reuse. Additionally, the long-term stability and environmental fate of these nanomaterials require further investigation to ensure safe and sustainable application.

#### 4.1.3. Antimicrobial Water Purification

Microbial contamination of water is a major threat to global public health, particularly in resource-limited settings, where contamination from agricultural, human, and animal waste is prevalent. Waterborne pathogens include bacteria (e.g., *Salmonella typhi*, *Escherichia coli*), viruses, fungi, algae, and protozoa, all of which compromise water quality and reuse. Despite their small size, effective removal of microbial contaminants often requires a combination of physical, chemical, and biological treatment methods, due to differences in microbial structure and resistance [68,69,70]. Conventional physical methods such as boiling and filtration are simple but may be limited in efficiency and scalability. Chemical and radiation-based treatments, including ozonation and UV-C irradiation, act by damaging microbial DNA/RNA, thereby preventing replication [71]. However, these approaches may not provide residual protection within water distribution systems, where recontamination can occur.

Phyto-mediated nanomaterials, particularly plant-derived silver nanoparticles (AgNPs) and certain metal oxide nanoparticles, have demonstrated strong antimicrobial activity against a broad spectrum of microorganisms [72,73]. The antimicrobial activity of PMNs is generally associated with cell-membrane disruption, ROS generation, and interference with intracellular metabolic processes. In addition, the presence of phytochemical residues on nanoparticle surfaces can enhance antimicrobial efficacy and improve biocompatibility, making these materials suitable for point-of-use water disinfection systems. For example, AgNPs synthesized using *Phaseolus vulgaris* (kidney bean) extracts demonstrated significant removal of reactive red-141 (RR-141) dye (~97% degradation within 150 min) alongside antibacterial activity against Gram-positive (*Bacillus subtilis*) and Gram-negative (*Escherichia coli*) bacteria, with inhibition zones of 15 and 18 mm, respectively [59].

Green-synthesized metal oxides further extend the antimicrobial potential of PMNs beyond Ag-based systems. ZnO nanoparticles synthesized using Cosmos caudatus extract exhibited antimicrobial activity against *S. aureus, E. coli*, and Aspergillus niger, with the strongest activity observed against *E. coli* [66]. Similarly, ZnO nanoparticles synthesized using Magnolia officinalis, Goldthread, and Lonicera japonica extracts showed strong and long-lasting antimicrobial activity against *E. coli, S. aureus*, and Botrytis cinerea [74]. ZnO nanoparticles synthesized using Syzygium aromaticum and Thymus capitatus extracts also exhibited broad antibacterial and antifungal activity, further supporting the versatility of plant-mediated ZnO as an antimicrobial material [75]. Plant-mediated ZrO_2_ also shows antimicrobial potential. Jasminum officinale-mediated ZrO_2_ nanoparticles inhibited the fungal pathogen Fusarium oxysporum, with the optimized JO-ZrO_2_ formulation showing the greatest fungal-growth inhibition [67]. Thus, phyto-mediated ZnO and ZrO_2_ provide complementary antimicrobial functionality and broaden the range of green-synthesized metal oxides available for environmental and water-related applications.

However, several challenges remain, including potential nanoparticle toxicity at higher concentrations, reduced antimicrobial efficiency in complex water matrices, and difficulties in recovery and reuse. Additionally, the long-term environmental impact and potential development of microbial resistance require further investigation.

#### 4.1.4. Synergistic Adsorption–Catalysis Mechanisms

One of the key advantages of PMNs is their ability to support synergistic pollutant removal mechanisms, where adsorption and catalytic or photocatalytic degradation occur either simultaneously or sequentially at the nanoparticle surface. In such systems, adsorption concentrates contaminants at reactive surface sites, while catalytic processes facilitate their transformation into less toxic or mineralized products. This synergy improves overall removal efficiency and reduces the need for multiple treatment steps. The synthesis of *Chlorella vulgaris* (algae)-decorated TiO_2_/Ag hybrid nanofiber membranes has been reported for the removal of Cr(VI) under light irradiation. Upon UV illumination, photo-generated electrons from the TiO_2_ conduction band participate in the reduction of Cr(VI) to Cr(III), while the presence of bio-derived components enhances charge separation and reduces electron–hole recombination. Simultaneously, organic pollutants undergo degradation via ROS generated during the photocatalytic process [76]. The surface functionality introduced by plant-derived capping agents plays a critical role in enabling this synergistic behavior, as functional groups such as –OH, –COOH, and phenolic moieties enhance adsorption while also facilitating electron transfer processes. This multifunctional capability is particularly advantageous for treating complex wastewater systems containing mixed inorganic, organic, and biological contaminants, where single-mechanism approaches are often insufficient. However, under mixed-contaminant conditions, adsorption and photocatalysis may also compete for available surface-active sites. Strongly adsorbed metal ions or organic species can block photocatalytically active sites and restrict access to other contaminants, whereas accumulated reaction intermediates may further suppress catalytic turnover. Conversely, adsorption can enhance photocatalytic degradation when it concentrates on target organic molecules near ROS-generating sites. Thus, the balance between synergistic and competitive interactions depends on contaminant composition, surface coverage, adsorption affinity, pH, and light intensity. Potential deactivation of active sites over time may further reduce treatment efficiency and limit the consistency of performance under real-world conditions.

### 4.2. Environmental Remediation

Human activities affect not only water systems but also soil, groundwater, and air, leading to continuous contamination that necessitates effective remediation strategies. Green nanotechnology leverages the diverse phytochemical composition of plant extracts for the synthesis of PMNs are increasingly applied in environmental remediation, particularly in contaminated soil and subsurface environments [77,78]. The unique combination of surface functionality, reactivity, and environmental compatibility of plant-derived nanomaterials makes them suitable for addressing complex pollutant mixtures in terrestrial and groundwater systems. In situ remediation approaches, contaminants treated directly within the affected environment minimize the need for excavation or extensive material handling.

PMNs are particularly well suited for such strategies due to their relatively low toxicity, biodegradable surface coatings, and reduced environmental footprint [79]. When introduced into contaminated soils or aquifers, these materials can participate in contaminant immobilization, catalytic transformation, and redox-driven degradation processes. In addition, their use in situ allows integration with natural attenuation and microbial processes, enhancing overall remediation efficiency. For example, TiO_2_ nanoparticles synthesized via plant-mediated routes can enhance the degradation of polyaromatic hydrocarbons by facilitating electron transfer processes and promoting both microbial and abiotic transformation pathways [80]. Despite their promising performance, several challenges remain, including limited control over nanoparticle transport and distribution in subsurface environments, potential interactions with natural organic matter that may reduce reactivity, and uncertainties regarding long-term environmental fate [81]. These limitations highlight the need for improved delivery strategies and comprehensive field-scale validation.

In natural environmental systems, the performance of nanomaterials is influenced by interactions with natural organic matter (NOM), including humic substances, fulvic acids, and dissolved organic carbon. PMNs often exhibit improved compatibility with NOM due to their biofunctionalized surfaces. The presence of plant-derived organic moieties can reduce nanoparticle aggregation and enhance colloidal stability, thereby preserving surface reactivity and accessibility of active sites [82]. At the same time, interactions with NOM can modify pollutant removal mechanisms by altering surface charge, redox behavior, and adsorption dynamics. However, excessive NOM may compete with target pollutants for active sites or block reactive surfaces, potentially reducing removal efficiency. Therefore, the interplay between PMNs and natural organic matter remains a critical factor in the design and application of sustainable remediation technologies [83,84].

#### Soil and Groundwater Remediation

Soil and groundwater contamination by heavy metals, hydrocarbons, pesticides, and industrial chemicals represents a significant environmental challenge due to the persistence and mobility of these pollutants in natural systems. Groundwater contamination by arsenic, nitrate, chlorinated solvents, and microbial pathogens remains a major global concern [85,86]. PMNs have attracted increasing attention for the immobilization, transformation, and removal of contaminants from soil and groundwater systems. Plant-derived functional groups present on nanoparticle surfaces enhance contaminant binding affinity and improve dispersion stability in subsurface environments. Metal and metal oxide nanoparticles such as Ag, CuO, NiO, and TiO_2_ have demonstrated the ability to generate ROS, enabling degradation of organic pollutants including Congo red and methylene blue dyes into less toxic products [87,88]. Rahman et al. highlighted the use of bio-based nanomaterials derived from biochar, cellulose, algae, and chitosan for arsenic remediation in groundwater systems in Bangladesh, one of the global hotspots of arsenic contamination [54,89]. These materials exhibited enhanced arsenic removal through adsorption and precipitation mechanisms facilitated by their natural surface functionality.

Phytonanomaterial may complement phytoextraction, an environmentally friendly remediation technique that uses plants to accumulate and remove heavy metals from contaminated soils and water systems. During phytoextraction, plant roots absorb metal ions from polluted soil or water, usually heavy metals by chelation via phytochelatins. The chelated metal ions are transported to aerial tissues for subsequent sequestration and detoxification [90,91]. Hyperaccumulator plants are particularly important in this process due to their exceptional tolerance to elevated metal concentrations. Similar to groundwater remediation, plant-mediated metal and metal oxide nanoparticles interact with contaminants in soil systems through adsorption, surface complexation, redox reactions, and photocatalytic degradation mechanisms, thereby reducing contaminant mobility and bioavailability [92].

Several studies have combined phytoextraction with nano-enabled remediation strategies for environmental remediation and climate-relevant applications. Francy et al. reported the use of neem-and mint-derived nano-zero-valent iron particles for enhanced remediation of Pb- and Ni-contaminated soils. Different plant systems exhibit varying selectivity toward specific metals, Shad et al. demonstrated the application of *Mentha piperita*-derived nano-zero-valent iron particles for the removal of dissolved agricultural contaminants including phosphate, ammonia, nitrate, lead, and chloride from contaminated Worcester and Birmingham canal waters [60,61]. These promising findings of decontamination by neem, mint and *Mentha piperita* nano-zero valent iron particles demonstrate an eco-friendly and sustainable environmental remediation of PMNs comparable to CNMs.

Although, different studies have reported the environmental remediation potentials of PMNs through pollutant removal and contaminant stabilization. Still, increasing attention is being directed toward their potential role in climate-related environmental processes, particularly carbon sequestration, greenhouse gas mitigation, and soil carbon stabilization. Owing to their unique surface chemistry, redox activity, and interactions with soil organic matter and microbial communities, PMNs may contribute not only to pollution control but also to long-term climate sustainability. Therefore, understanding their role in carbon cycling and climate-relevant applications represents an important extension of their environmental functionality.

### 4.3. Carbon Sequestration and Climate-Relevant Applications of Phyto Mediated Nanomaterials

Despite global efforts to reduce greenhouse gas emissions, the continued increase in atmospheric carbon levels necessitates the development of innovative and sustainable mitigation technologies. In this context, PMNs have attracted increasing attention due to their potential applications in carbon sequestration and climate-related environmental remediation [93]. In addition to pollutant removal, PMNs can contribute to climate mitigation through photocatalytic degradation, CO_2_-conversion, mineralization, and stabilization of soil organic carbon (SOC). Photocatalytic degradation of organic pollutants generally proceeds via ROS generated at nanoparticle surfaces according to the following simplified reaction:

$$Organic\;poollutant+h^{+}/OH\to CO_{2}+H_{2}O+mineral\;acids$$

Plant-derived nanomaterials synthesized from biomass resources offer sustainable pathways for carbon capture, storage, and stabilization while simultaneously addressing environmental contamination [62]. These materials enhance soil carbon sequestration by improving soil aggregation, promoting mineral–organic associations, modulating microbial carbon-use efficiency, and reducing carbon mineralization rates, thereby stabilizing SOC. Iron-based nanomaterials exert additional control over soil carbon dynamics through coupled physicochemical and microbially mediated mechanisms. At the nanoscale, Fe(III)/Fe(II) oxides and nano-zero-valent iron (nZVI) possess high densities of surface hydroxyl groups capable of forming strong interactions with carboxylate and phenolic moieties of soil organic matter (SOM) [94]. These Fe–organic associations reduce enzymatic accessibility and suppress decomposition kinetics, thereby enhancing carbon stabilization. In addition, redox-active iron phases can function as electron shuttles that influence microbial respiration pathways and alter terminal electron-acceptor availability, potentially reducing oxidative mineralization and cumulative CO_2_ emissions. In composting and soil amendment systems, iron-based nanomaterials are reported to decrease greenhouse gas emissions while increasing humification indices, suggesting enhanced conversion of labile carbon into more stable mineral-associated fractions [95].

The SOC quality of the soil is an important factor in carbon lock by the plant root and root exudates from dynamic interactions among the soil microbes, roots and the rhizosphere. The SOC dynamics is commonly represented using multi-pool first-order decay models consisting of fast- and slow-cycling carbon pools characterized by decomposition constants (k_1_ and k_2_). Mineral interactions, including iron-mediated sorptive protection, can reduce the decomposition rate of labile carbon and increase soil carbon mean residence time, thereby enhancing long-term stabilization in mineral-associated organic matter pools. Because cumulative CO_2_ release is directly linked to decomposition kinetics, reductions in decomposition constants are expected to decrease seasonal carbon mineralization losses [96].

Despite these promising findings, the economic feasibility of biomass-based PMNs for large-scale carbon sequestration remains insufficiently evaluated. Although plant-derived feedstocks are often considered low-cost, large-scale production may involve hidden costs associated with biomass collection and transportation, drying and storage, extract preparation and purification, process standardization, and management of residual biomass. These additional requirements may partially offset the economic advantages generally attributed to phyto-mediated synthesis and should therefore be incorporated into techno-economic and life-cycle assessments. Moreover, uncertainties remain regarding the long-term environmental persistence, ecological impacts, and field-scale carbon sequestration performance of PMNs. Although recent studies support their potential for SOC stabilization and greenhouse-gas mitigation, field-scale evidence under realistic environmental conditions remains limited, emphasizing the need for long-term field trials to validate laboratory-scale observations. In addition, complex interactions between nanoparticles, microbial communities, and natural soil constituents remain insufficiently understood. Therefore, comprehensive long-term and field-scale studies are required before large-scale environmental deployment [97,98].

#### 4.3.1. Plant-Derived Biochar Nanomaterials

Biochar derived from plant biomass has emerged as a versatile carbonaceous material with promising applications in environmental remediation and carbon sequestration. When engineered into nano- or micro-structured forms, biochar materials exhibit high surface area, porous architecture, and abundant oxygen-containing functional groups that enhance adsorption and interfacial reactivity [99]. These physicochemical properties enable efficient removal of both organic and inorganic contaminants while simultaneously contributing to long-term carbon stabilization in environmental systems. The renewable origin, low-cost production, and relative chemical stability of plant-derived biochar nanomaterials make them particularly attractive for sustainable remediation and climate-relevant applications. In addition, surface functional groups such as hydroxyl, carboxyl, and phenolic moieties improve pollutant-binding affinity and may facilitate catalytic or electron-transfer processes during remediation.

The study by Kumari et al. demonstrated the application of *Murraya koenigii*-derived biochar encapsulated on lanthanum ferrite (BC/LaF) for ciprofloxacin remediation from contaminated water systems. The developed BC/LaF composite exhibited high adsorption capacity (98.86 mg g^−1^), highlighting the potential of plant-derived biochar nanomaterials for antibiotic removal. Compared with tannic acid-functionalized magnetite nanoparticles (TA/Fe_3_O_4_ NPs), which exhibited a lower adsorption capacity under the reported conditions (4.27 mg g^−1^), the BC/LaF system demonstrated enhanced adsorption performance [63]. However, adsorption efficiency in such systems is influenced by synthesis conditions, pollutant concentration, surface chemistry, and solution pH. Thus, making direct comparison between different nanomaterial systems challenging. Despite these advantages, plant-derived biochar nanomaterials still face several limitations, including variability in biomass feedstocks, inconsistency in pore structure and surface functionality, and uncertainties regarding long-term environmental stability and regeneration performance. Furthermore, large-scale production and standardization remain significant challenges that may limit practical environmental deployment.

#### 4.3.2. Carbon Capture and Storage Mechanisms

Plant-derived carbonaceous nanomaterials emerge as sustainable materials for carbon capture and storage (CCS) through multiple physicochemical and biologically mediated mechanisms. Proposed mechanisms involved in carbon stabilization include adsorption, photoreduction, mineral association, and microbial influenced carbon transformation pathways [100,101]. Carbonaceous PMNs may contribute to atmospheric CO_2_ capture through physical adsorption within porous structures and through surface-mediated chemical interactions involving oxygen-containing functional groups [102,103]. In soil and sediment systems, biochar-based nanomaterials can persist for extended periods, supporting long-term carbon sequestration and reducing carbon re-release into the atmosphere. In addition, nanoparticles such as ZnO, SiO_2_, and Fe_3_O_4_ may promote the formation of stable soil microaggregates by bridging soil particles and organic matter, thereby improving physical protection of SOC. Functionalized biochar nanomaterials can also enhance native SOC retention by increasing sorptive capacity and reducing carbon mineralization rates [104].

Kavya et al. provided a comprehensive assessment of physical, chemical, and biological mechanisms through which agricultural soils function as carbon sinks [105]. Their review highlighted the role of iron oxide- and clay-based nanoparticles in stabilizing organic carbon through strong interactions with organic acids, humic substances, and root-derived compounds. These interactions may reduce carbon mobility and improve long-term SOC stabilization. In addition, certain nanomaterials may influence catalytic and redox processes associated with CO_2_ conversion and suppression of excessive enzymatic degradation, thereby contributing to climate-relevant carbon retention pathways [106,107]. Despite these promising mechanisms, important limitations remain, including uncertainties regarding long-term nanoparticle stability, interactions with soil microbial communities, and the environmental consequences of nanoparticle accumulation in terrestrial systems. Furthermore, most current studies remain laboratory-scale, highlighting the need for field validation and life-cycle assessment before practical large-scale implementation.

#### 4.3.3. Role in Mitigating Greenhouse Gas Emissions from Agriculture

Carbon dioxide (CO_2_), methane (CH_4_), and nitrous oxide (N_2_O) are major greenhouse gases emitted during agricultural activities, including composting, fertilizer application, and intensive soil management practices. These emissions contribute significantly to global warming and climate change. Recent study by Youns et al. demonstrated that the application of nanomaterials in carbon dioxide storage play important role in mitigating climate impact from emission of greenhouse gas. In addition, nanoparticles such as TiO_2_ and SiO_2_ are helpful in carbon sink through regulation of microbial activity, nutrient cycling, and redox processes between the plant roots and agricultural soils [108]. Wang et al. indicated that ZnO nanoparticles successfully reduced N_2_O emissions in tea plantation soils by approximately 33% at a concentration of 100 mg kg^−1^, showing greater mitigation efficiency than Fe_3_O_4_ nanoparticles. Considering that N_2_O possesses a global warming potential nearly 300 times greater than CO_2_, such reductions are environmentally significant [109].

Similarly, Tian et al. demonstrated that iron-based nanomaterials incorporated into composting systems reduced CO_2_, CH_4_, and N_2_O emissions compared with untreated controls, highlighting their potential role in lowering greenhouse gas emissions during organic waste management. In addition to iron- and zinc-based nanomaterials, other metal oxide nanoparticles are being investigated for greenhouse gas mitigation [94]. Nitrous oxide emission is a major greenhouse gas from agricultural land due to application of fertilizers, manure, crop residues, and microbial transformation of nitrogen in the soil. Study from Hu et al. showed that Al_2_O_3_ nanoparticles reduced soil N_2_O emissions by suppressing nitrification processes, whereas CuO nanoparticles influenced nitrification and denitrification functional genes, resulting in variable effects on N_2_O release from soils [110].

Despite these promising findings, studies specifically investigating the role of PMNs in greenhouse gas mitigation remain limited. Nevertheless, plant-derived nanomaterials may indirectly contribute to emission reduction by improving soil quality, enhancing nutrient retention, stabilizing soil organic carbon, and modulating microbial processes associated with CO_2_, CH_4_, and N_2_O production. In agricultural and contaminated land systems, plant-derived biochar nanomaterials have been associated with reduced greenhouse gas fluxes, improved carbon stability, and enhanced soil resilience [111]. Furthermore, Mridha et al. discussed the application of synthesized smart nanoparticles from phyto waste as sustainable tools for greenhouse gas monitoring and mitigation in eco-friendly agricultural systems [112]. With the considerable potential for climate-relevant agricultural applications, there is need for more research to validate nanoparticle–microbe–soil interactions and the large-scale implementation for sustainable farming systems.

### 4.4. Comparative Analysis: Conventional vs. Phyto-Mediated Nanomaterials

A critical assessment of environmental nanotechnology requires direct comparison between CNMs and phyto-mediated systems. Conventional synthesis involves classical methods, including chemical reduction using sodium borohydride (NaBH_4_), hydrothermal synthesis, and physical vapor deposition, provide precise control over nanoparticle size, morphology, crystallinity, and monodispersity. However, these approaches often rely on toxic reducing agents, organic solvents, and non-biodegradable stabilizers that may contribute to secondary environmental pollution and increased ecological risk [113].

In contrast, PMNs are characterized by the presence of a biogenic surface corona composed of plant-derived phytochemicals such as polyphenols, proteins, carbohydrates, and flavonoids. This organic layer functions not only as a reducing and stabilizing agent during synthesis but also contributes additional reactive sites for adsorption and redox-mediated remediation processes [114]. For example, polyphenolic ligands associated with biogenic Ag–Fe nanoparticles synthesized using neem leaf extracts may provide supplementary redox-active and metal-binding functionalities that are often absent in the conventionally synthesized counterparts [115]. Furthermore, conventional nanoparticles frequently require post-synthesis functionalization to improve colloidal stability and prevent aggregation under environmentally relevant conditions, particularly in high-salinity or complex aqueous systems. By contrast, PMNs often exhibit improved dispersion stability and biocompatibility due to natural steric stabilization provided by plant-derived biomolecules adsorbed on the nanoparticle surface [116].

Among widely investigated metal oxide nanomaterials, ZnO, TiO_2_, and ZrO_2_ represent important platforms for environmental remediation because of their surface reactivity, chemical stability, and photocatalytic properties. Plant-mediated synthesis provides a greener route for producing these oxides while retaining their environmental functionality. Green-synthesized ZnO has demonstrated substantial photocatalytic and antimicrobial performance [66,74,75]. Similarly, TiO_2_ nanoparticles synthesized using Inula viscosa leaf extract achieved complete degradation of methylene blue and tartrazine within 60 min under UV irradiation, demonstrating the potential of phyto-mediated TiO_2_ for organic pollutant removal [117]. Plant-mediated ZrO_2_ has also demonstrated effective photocatalytic degradation of organic dyes together with antimicrobial activity [67]. Collectively, these studies demonstrate that phyto-mediated synthesis is applicable not only to metallic nanoparticles but also to widely used semiconductor oxides such as ZnO, TiO_2_, and ZrO_2_, broadening their potential for sustainable environmental remediation.

As summarized in Table 1, the performance gap between conventional and PMNs is progressively narrowing. Although conventionally synthesized nanoparticles may exhibit higher surface area and greater structural uniformity due to controlled synthesis conditions, PMNs often demonstrate enhanced selectivity and surface functionality. In heavy metal remediation systems, multifunctional surface ligands such as hydroxyl and carboxyl groups can promote stronger surface complexation and improved affinity toward Pb^2+^ and Cd^2+^ ions compared with unmodified synthetic oxides [40,118]. Nevertheless, several limitations continue to constrain the large-scale application of phyto-mediated nanomaterials. One of the major challenges is the variability in phytochemical composition among plant species, growth conditions, harvesting seasons, and geographical locations. Such heterogeneity can significantly influence nanoparticle synthesis, surface chemistry, and remediation performance, thereby limiting reproducibility and industrial standardization. Unlike highly purified industrial chemicals used in conventional synthesis routes, plant extracts represent complex and variable biological systems. Consequently, future development of phyto-mediated nanotechnology may require the use of standardized plant-derived molecular fractions and improved process control strategies to ensure consistent large-scale performance.

## 5. Mechanistic Insights into Pollutant Removal

**Figure 4:**
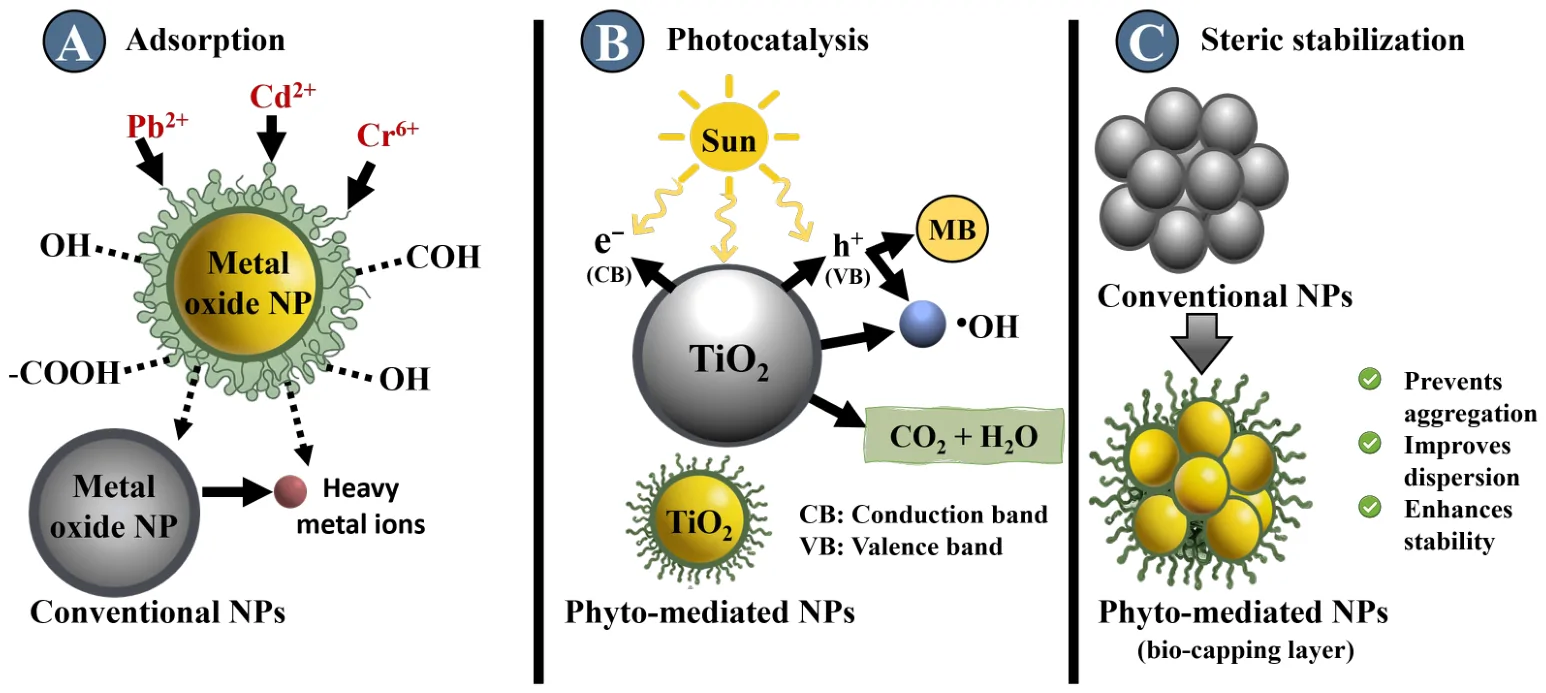
Major remediation mechanisms of PMNs: (**A**) phytochemical functional groups promote heavy-metal adsorption and surface complexation; (**B**) phytochemical-assisted charge transfer facilitates photocatalytic pollutant degradation; and (**C**) the biogenic corona provides steric stabilization, reducing aggregation and improving nanoparticle dispersion.

Industrial activities including textile manufacturing, dye processing, and pharmaceutical production generate large volumes of contaminated wastewater containing dyes, antibiotics, pharmaceutical residues, and other persistent organic pollutants that disrupt environmental and ecological systems. The effectiveness of PMNs in remediation applications is governed by the physicochemical mechanisms through which they interact with contaminants. These mechanisms are influenced not only by nanoparticle size, composition, and surface area, but also by the bioactive phytochemicals introduced during plant-mediated synthesis [119]. The major remediation mechanisms associated with PMNs, including adsorption, photocatalytic degradation, and steric stabilization, are schematically illustrated in Figure 4. Together, these pathways illustrate how the phytochemical surface layer can influence pollutant binding, photoinduced transformation, and nanoparticle dispersion, thereby linking surface chemistry to remediation performance.

PMNs remove pollutants through multiple interconnected pathways, including adsorption, photocatalytic degradation, redox transformation, microbial stimulation, and rhizosphere-assisted interactions. The biogenic corona of PMNs can modify their interfacial interactions with contaminants through phytochemical functional groups, such as hydroxyl (–OH), carboxyl (–COOH), and amine (–NH_2_) moieties. As illustrated in Figure 4A, these functional groups provide binding sites for adsorption and surface complexation with heavy metal ions such as Pb^2+^ and Cd^2+^ [120,121]. Thus, Panel A demonstrates the direct relationship between phytochemical surface functionality and contaminant binding.

Plant-derived surface components may also influence the photocatalytic behavior of semiconductor PMNs. As illustrated in Figure 4B, biomolecules such as chlorophylls, flavonoids, and carotenoids may function as photosensitizers by absorbing visible light and facilitating electron transfer into the conduction band of semiconductor materials such as TiO_2_. This mechanism may extend the photoresponse toward the visible-light region, whereas many conventional semiconductor photocatalysts primarily rely on UV activation [122,123,124]. Panel B therefore links phytochemical-assisted charge transfer with photocatalytic pollutant degradation.

The biogenic corona can additionally influence the colloidal stability and transport behavior of PMNs in environmental matrices. Surface-bound phytochemicals may provide steric stabilization that limits aggregation and maintains nanoparticle dispersion under suitable environmental conditions. As schematically illustrated in Figure 4C, these interactions may affect nanoparticle mobility, accessibility of active sites, and persistence of reactivity during remediation [125]. This panel therefore highlights how steric stabilization can influence the availability and transport of PMNs in environmental media.

However, these effects may change as PMNs undergo environmental aging. The stability of the biogenic corona under varying environmental conditions, transformation of phytochemical surface species, and interactions with natural organic matter and microbial communities remain insufficiently understood. The intrinsic biodegradability of phytochemical surface layers may progressively alter or remove the biogenic corona, resulting in changes in surface chemistry, colloidal stability, and remediation performance over time [126]. Such degradation may therefore contribute to environmental aging and functional decline of PMNs under long-term exposure conditions. Furthermore, the relative contributions of individual remediation pathways, including adsorption, photocatalysis, redox transformation, and microbial interactions, often remain difficult to distinguish in complex environmental systems. Further mechanistic and field-scale investigations are therefore required to determine how these pathways evolve under environmentally relevant conditions.

### 5.1. Adsorption and Surface Complexation

Adsorption is one of the principal mechanisms through which PMNs remove contaminants from water, soil, and groundwater systems. The exceptionally high surface-area-to-volume ratio of nanoscale materials provides abundant active sites for pollutant interaction and retention. In phyto-mediated systems, plant-derived capping agents introduce functional groups such as hydroxyl, carboxyl, amine, and phenolic moieties onto nanoparticle surfaces, facilitating pollutant removal through electrostatic attraction, hydrogen bonding, π–π interactions, and surface complexation mechanisms. Through these interactions, a wide range of contaminants including pesticides, dyes, pharmaceutical residues, and organic pollutants can be effectively immobilized or removed from aqueous and soil environments. For heavy metal ions, coordination interactions between surface functional groups and dissolved metal species play a dominant role in adsorption, thereby reducing metal mobility, toxicity, and bioavailability [127].

### 5.2. Photocatalytic and Redox Processes

Plant-mediated nanomaterials, particularly semiconductor systems such as TiO_2_, ZnO, CuO, and their composites, play important roles in photocatalytic and redox-driven pollutant transformation processes. Upon exposure to light irradiation or suitable redox conditions, these nanomaterials generate ROS and electron–hole (e^−^/h^+^) pairs capable of degrading organic contaminants into less toxic or mineralized products [128]. The presence of phytochemical residues on nanoparticle surfaces can significantly influence catalytic performance by improving visible-light absorption, facilitating interfacial charge transfer, and suppressing electron–hole recombination. As a result, phyto-mediated photocatalysts may exhibit enhanced catalytic efficiency compared with conventionally synthesized counterparts under environmentally relevant conditions. In addition to photocatalytic degradation, redox-active nanomaterials can promote oxidation–reduction reactions involving chlorinated compounds, dyes, pharmaceutical residues, and toxic metal species such as Cr(VI), thereby contributing to pollutant detoxification, immobilization, or transformation [129]. However, several limitations remain, including reduced photocatalytic efficiency in complex environmental matrices, catalyst deactivation, and incomplete understanding of long-term nanoparticle transformation pathways under natural environmental conditions.

### 5.3. Antimicrobial Mechanisms

PMNs exhibit antimicrobial activity through multiple concurrent mechanisms that contribute to water disinfection and pathogen control. These mechanisms include disruption of microbial cell membranes, generation of reactive oxygen species, interference with enzymatic and metabolic pathways, and damage to nucleic acids and intracellular biomolecules. A review by Lithi et al. highlighted the application of plant-mediated metal nanoparticles (Ag, Cu, and Au) and metal oxide nanoparticles (ZnO, MgO, Co_3_O_4_, and TiO_2_) for antimicrobial applications. The synergistic effects between metallic cores and bioactive phytochemicals contribute to broad-spectrum antimicrobial performance against bacteria, fungi, and certain viruses, making PMNs promising candidates for water purification and environmental disinfection applications [130,131].

### 5.4. Mechanistic Remediation Performance Across Pollutant Classes

The remediation performance for a specific contaminant requires integrating pollutant-specific removal pathways rather than evaluating nanomaterials solely according to material composition. Different contaminant classes interact with nanomaterials through distinct physicochemical mechanisms that ultimately govern removal efficiency, selectivity, transformation products, and long-term environmental stability.

#### 5.4.1. Heavy Metals

For heavy metal contaminants, remediation is primarily governed by adsorption, inner-sphere surface complexation, ion exchange, and redox transformation processes. Iron-based nanomaterials are particularly effective in reducing toxic Cr(VI) to the less mobile and less toxic Cr(III), while surface hydroxyl groups on metal oxides facilitate coordination interactions with Pb^2+^, Cd^2+^, As species, and other dissolved metal ions. In phyto-mediated systems, plant-derived phytochemicals introduce additional carboxyl, hydroxyl, and phenolic groups that can enhance chelation and adsorption affinity. However, quantitative adsorption constants and long-term stability data for these interactions remain insufficiently reported. Surface charge, solution pH, ionic strength, and competing ions strongly influence selectivity and overall removal performance under realistic environmental conditions [132,133,134,135].

#### 5.4.2. Organic Micropollutants

Organic contaminants such as pharmaceuticals, dyes, pesticides, and endocrine-disrupting compounds can be removed through photocatalytic oxidation, redox reactions, electron transfer pathways, and π–π adsorption interactions. Semiconductor nanomaterials generate ROS, including hydroxyl radicals and superoxide species, which initiate oxidative cleavage of aromatic rings and other functional groups. Carbon-based nanomaterials additionally enhance adsorption through π–π stacking interactions and hydrophobic effects. Despite the high degradation efficiency often reported under laboratory conditions, complete mineralization is not always achieved, and potentially toxic intermediate products may persist. Consequently, identifying degradation pathways and evaluating transformation-product toxicity remain important challenges for practical environmental implementation [136,137,138,139].

#### 5.4.3. Nutrients and Emerging Contaminants

Nutrient contaminants such as nitrates and phosphates are removed through adsorption-mediated immobilization or redox conversion mechanisms. In phyto-mediated systems, removal efficiency depends strongly on the chemical affinity between nanoparticle surfaces and oxyanion species. For phosphate removal, ligand exchange reactions and inner-sphere complex formation on Fe- and Al-based surfaces provide relatively stable sequestration mechanisms that are less sensitive to competing ions [140]. Emerging contaminants such as per- and polyfluoroalkyl substances (PFAS) present substantially greater remediation challenges due to the exceptional stability of the C–F bond (~485 kJ mol^−1^). Conventional photocatalytic systems often fail to achieve complete mineralization of these compounds. Consequently, recent research has focused on multifunctional nanohybrids that combine adsorption-driven concentration mechanisms with advanced photocatalytic or redox-active semiconductor systems capable of initiating defluorination reactions. In some cases, phyto-mediated surface coatings may additionally improve colloidal stability and reduce dissolution of reactive metal cores under harsh operational conditions. Nevertheless, large-scale application of such systems remains at an early developmental stage [141,142].

#### 5.4.4. Air Pollutants

Nanomaterial-based remediation systems have also demonstrated potential for the treatment of gaseous pollutants and airborne contaminants. Photocatalytic coatings containing semiconductor nanomaterials can oxidize volatile organic compounds (VOCs), nitrogen oxides (NOx), and sulfur-containing pollutants under ambient light conditions. The high surface-to-volume ratio of nanoscale catalysts enhances interactions between gaseous molecules and reactive surface sites, facilitating oxidation reactions that convert pollutants into less harmful species such as nitrates and sulfates [143]. In addition, incorporation of nanomaterials into construction materials and filtration systems has been explored for reducing urban air pollution and secondary aerosol formation. However, the practical efficiency of these systems remains strongly dependent on environmental conditions including humidity, pollutant concentration, airflow dynamics, and light intensity. In particular, reduced photocatalytic performance under low-light or indoor conditions remains a significant limitation, motivating ongoing efforts toward the development of visible-light-responsive and narrow-bandgap nanomaterials [144,145,146,147].

## 6. Environmental Safety, Toxicity, and Risk Assessment

Compared with conventionally synthesized nanomaterials, phyto-mediated systems are considered more environmentally compatible because they avoid the use of highly toxic reducing agents and often possess biodegradable surface coatings derived from plant metabolites. Conventional synthesis routes frequently rely on energy-intensive processes and hazardous chemicals, increasing the overall environmental burden throughout the material life cycle [148]. However, despite their sustainability advantages, PMNs still face important challenges related to reproducibility, variability in plant extract composition, long-term stability, and scalability. Consequently, careful optimization and standardized evaluation remain necessary to ensure consistent environmental and functional performance. Although PMNs have demonstrated promising remediation capabilities, their environmental safety and potential ecological risks require thorough assessment before large-scale deployment. Most phyto-mediated systems still contain inorganic metallic or metal oxide cores such as Ag, Ni, Cu, Au, ZnO, and Fe-based nanostructures, which may undergo transformation, dissolution, or accumulation under environmental conditions [146]. Hence, understanding the fate, transport behavior, persistence, and ecological interactions of these materials is essential to ensure that remediation processes do not generate secondary environmental risks [149,150].

### 6.1. Ecotoxicological Considerations

The ecotoxicity of nanomaterials are influenced by multiple physicochemical factors including particle size, morphology, crystallinity, surface chemistry, concentration, aggregation behavior, and exposure duration. PMNs are often regarded as less toxic than chemically synthesized counterparts because plant-derived surface coatings may reduce direct surface reactivity and minimize the presence of hazardous synthetic residues. Nevertheless, these materials can still interact with aquatic and terrestrial organisms, potentially affecting microbial communities, plants, invertebrates, and higher trophic levels. Sub-lethal responses such as oxidative stress, membrane damage, enzymatic inhibition, metabolic disruption, and altered gene expression remain important considerations in ecotoxicological evaluation [151].

Beyond direct cellular toxicity, the environmental behavior of phyto-mediated nanoparticles is also influenced by secondary interactions occurring after environmental release. Once introduced into soil or aquatic systems, nanoparticles may acquire additional organic or inorganic surface layers that modify their mobility, bioavailability, and ecological interactions. Although phytochemical coatings can reduce excessive ROS generation, long-term persistence and trophic transfer may still lead to bioaccumulation concerns. In some cases, nanoparticle surfaces may facilitate the co-transport of adsorbed contaminants across biological membranes, a phenomenon commonly referred to as the “Trojan horse” effect. Consequently, future ecotoxicological studies should move beyond short-term mortality assessments toward integrated omics-based approaches capable of evaluating genomic, proteomic, and metabolomic responses in non-target organisms. Such approaches may provide a more comprehensive understanding of potential ecosystem-level impacts, including alterations in microbial diversity and nutrient cycling processes [152,153].

### 6.2. Environmental Fate and Transport

PMNs and waste-derived nanobiochar systems may contribute positively to circular-economy strategies and sustainable environmental remediation. However, once released into environmental systems, these materials can undergo aggregation, dissolution, oxidation, surface transformation, and interactions with natural organic matter, dissolved ions, minerals, and biological components. Such processes strongly influence nanoparticle mobility, persistence, bioavailability, and remediation efficiency under realistic environmental conditions [154]. Plant-derived surface coatings may improve colloidal stability and dispersion behavior, but they can also alter transport dynamics within soil and groundwater systems. Therefore, standardized operational parameters, including dosage, exposure time, environmental pH, ionic strength, and organic matter content, remain essential for predicting nanomaterial distribution, longevity, and reactivity in environmental applications.

The transport behavior of phyto-mediated nanoparticles is governed by the balance between particle attachment, electrostatic interactions, steric stabilization, and aggregation kinetics. In porous subsurface environments such as aquifers, the phytochemical corona may act similarly to a natural surfactant, reducing nanoparticle deposition onto mineral surfaces including silica and alumina. However, variations in ionic strength, multivalent ions, and pH conditions may destabilize the colloidal system and promote hetero-aggregation. For nanobiochar systems, transport behavior may be further complicated by low density, irregular morphology, and interactions with mobile soil colloids, which can facilitate the co-transport of adsorbed heavy metals and hydrophobic organic contaminants over considerable distances [155,156]. Despite increasing laboratory-scale investigations, substantial uncertainties remain regarding the long-term environmental persistence, transformation pathways, and field-scale transport behavior of phyto-mediated nanomaterials. Future studies should therefore integrate laboratory experiments, mesocosm systems, and real environmental monitoring to better assess environmental risks and establish reliable regulatory frameworks for safe implementation.

### 6.3. Bioaccumulation and Trophic Transfer

The potential for bioaccumulation and trophic transfer of PMNs remains an active area of investigation. Biodistribution of nanomaterials in and trophic transfer in aquatic system depend on the physicochemical parameters of the phyto-nanomaterials including the size, shape, and concentrations. Although biogenic surface functionalization of phyto-metallic nanomaterials may reduce nanoparticle bioavailability and acute toxicity, microorganisms, plants, and aquatic organisms can take them up, with possible transfer across food webs [157]. Consequently, evaluating accumulation patterns and long-term ecological consequences is essential, particularly for applications involving repeated or large-scale environmental deployment. Recent studies have demonstrated that soil bacteria, fungi, and plant roots through endocytosis-like pathways or direct penetration of root epidermal tissues may internalize metal and metal oxide nanoparticles such as Ag, ZnO, and CuO. Once internalized, nanoparticles can undergo physicochemical transformations including dissolution, oxidation, and sulfidation, which may alter their mobility, persistence, and biological effects. In plants, translocation from roots to aerial tissues through xylem transport has been reported, raising concerns regarding entry into herbivorous trophic levels and subsequent ecological transfer. Additionally, interactions between nanoparticles and natural organic matter or biomolecules may either suppress or enhance bioavailability depending on environmental conditions such as pH, ionic strength, and microbial activity [158].

Trophic transfer has already been observed in simplified aquatic and terrestrial model systems, where nanoparticles accumulated in primary producers were subsequently detected in invertebrates and higher-level consumers. Even when acute toxicity appears limited, uncertainties remain regarding chronic exposure, long-term retention, and biomagnification potential. Therefore, future studies should incorporate long-term mesocosm experiments, isotopic tracing approaches, and life-cycle-based risk assessment frameworks to better evaluate the ecological safety of phyto-mediated remediation technologies [159,160].

### 6.4. Transformation and Fate in Aquatic Systems

Metal- and metal oxide-based nanomaterials released during remediation processes may enter surrounding aquatic environments, creating potential exposure pathways for both ecosystems and human populations. Once introduced into water systems, conventionally synthesized and phyto-mediated nanoparticles undergo a series of complex physicochemical and biological transformations. Environmental parameters including pH, ionic strength, temperature, dissolved oxygen, and natural organic matter (NOM) strongly influence nanoparticle stability, aggregation behavior, dissolution, and surface speciation. For example, elevated ionic strength may compress the electrical double layer surrounding nanoparticles, promote aggregation and sedimentation while reduce colloidal mobility. Conversely, interactions with NOM can enhance steric or electrostatic stabilization, thereby increasing nanoparticle persistence and transport distances within aquatic systems.

Dissolution processes are particularly important for Ag, ZnO, and CuO nanoparticles because released metal ions often govern both toxicity and antimicrobial activity. Similarly, iron-based nanoparticles may undergo oxidation to Fe(II)/Fe(III) oxides or hydroxides, altering their reactivity and contaminant-binding properties over time. In phyto-mediated systems, plant-derived capping agents such as polyphenols, flavonoids, and proteins may initially improve colloidal stability and reduce acute toxicity. However, these organic surface coatings can gradually undergo photodegradation, microbial degradation, or competitive displacement in natural waters, leading to changes in surface charge, aggregation behavior, and environmental reactivity. The progressive loss or transformation of these bio-capping layers may also reduce the availability of phytochemical functional groups involved in pollutant adsorption, surface complexation, and interfacial electron transfer, thereby contributing to a gradual decline in remediation performance during long-term environmental exposure. Such transformations directly influence nanoparticle bioavailability, trophic transfer potential, and sediment accumulation behavior [161,162]. Despite increasing laboratory-scale investigations, important uncertainties remain regarding long-term persistence, transformation kinetics, and environmental behavior under realistic field conditions. Additional studies integrating environmental monitoring, mesocosm systems and long-term exposure assessment are therefore required to understand the fate of PMNs in aquatic ecosystems.

### 6.5. Risk Assessment and Regulatory Perspectives

Comprehensive risk assessment frameworks are essential for evaluating the environmental and human-health implications of phyto-mediated nanomaterials. Such assessments should consider physicochemical properties, exposure pathways, environmental persistence, transformation behavior, life-cycle impacts, regional environmental conditions, and end-of-life disposal scenarios. At present, regulatory frameworks specifically designed for nanomaterials remain limited. In many countries, nanomaterials are still regulated under conventional chemical legislation despite their distinct nanoscale behavior and environmental interactions. Existing regulatory systems including frameworks established by the United States Environmental Protection Agency (EPA), the European Union REACH legislation, the Canadian Environmental Protection Act (CEPA), and related international regulatory agencies primarily emphasize material characterization, production, handling, exposure control, and disposal practices [163,164]. However, these regulations often do not fully address the unique surface properties, transformation pathways, mobility characteristics, and biological interactions associated with nanoscale materials, particularly phyto-mediated systems possessing dynamic biogenic surface coatings. Furthermore, the absence of standardized toxicity-testing protocols and harmonized environmental assessment methodologies complicates cross-study comparisons and regulatory implementation. Therefore, the development of dedicated testing guidelines, standardized characterization methods, and nano-specific environmental risk assessment models remains critical for the safe, responsible, and sustainable application of PMNs in environmental remediation technologies [165].

## 7. Current Challenges and Knowledge Gaps

**Figure 5:**
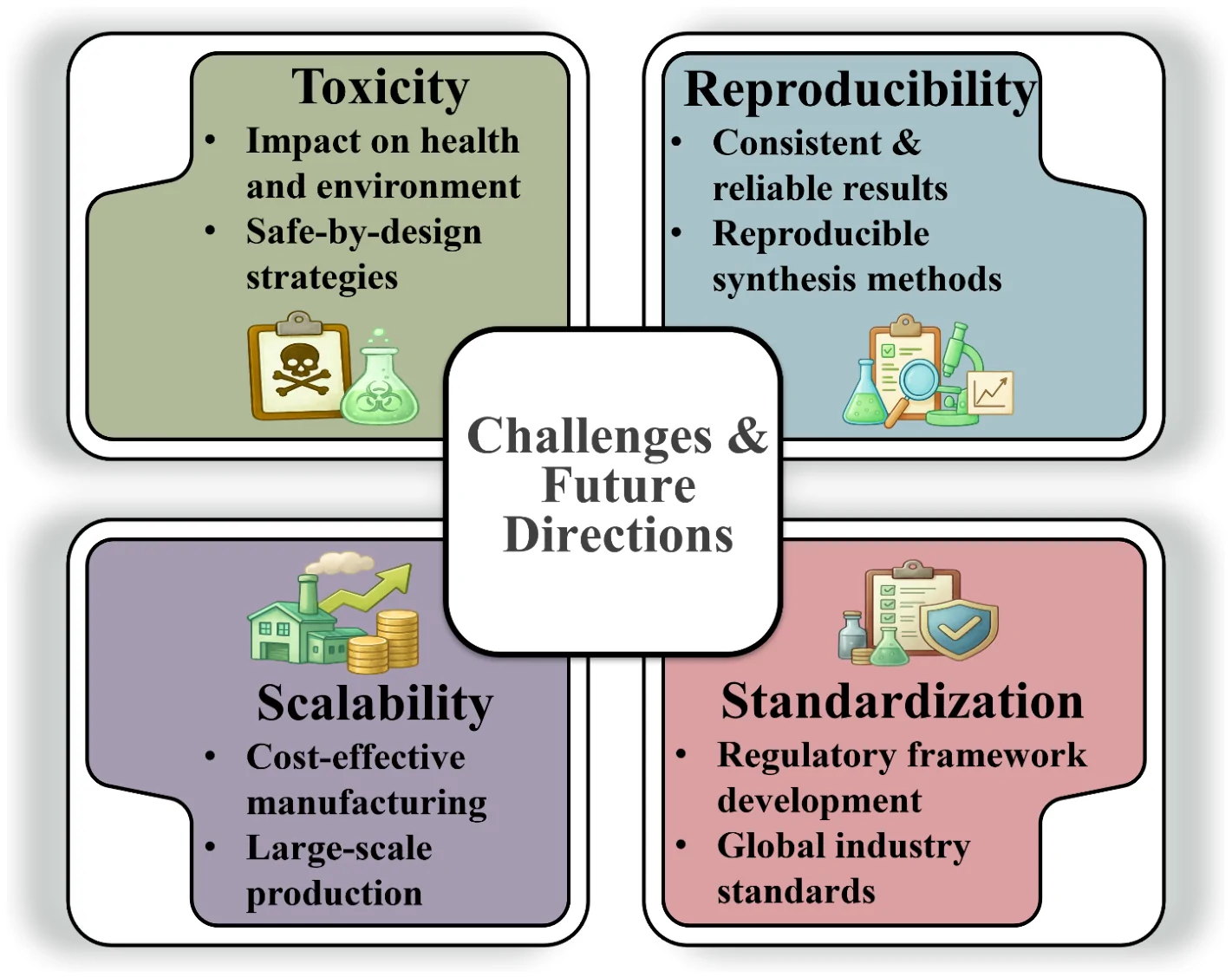
Major challenges and future directions for PMNs in environmental remediation, highlighting the need to address toxicity, reproducibility, scalability, and standardization to support their safe, consistent, and large-scale implementation.

Despite the growing interest in PMNs for environmental applications, several scientific, technical, environmental, and regulatory challenges remain unresolved. Addressing these limitations is essential for translating plant-based nanomaterials from laboratory-scale studies into reliable, scalable, and commercially viable environmental technologies. As summarized in Figure 5, major challenges include limited reproducibility, variability in plant extract composition, scale-up constraints, insufficient mechanistic understanding, long-term environmental uncertainty, and the absence of standardized regulatory frameworks. Accordingly, future efforts should prioritize safe-by-design strategies, reproducible synthesis protocols, cost-effective scale-up, and standardized regulatory frameworks to support the reliable and responsible implementation of PMNs.

One of the most significant limitations associated with Phyto-mediated nanochemistry is the reproducibility of nanoparticle synthesis. Plant-mediated processes inherently depend on biological systems with highly variable chemical compositions. Differences in extraction procedures, precursor concentrations, reaction conditions, and environmental growth factors can lead to inconsistencies in nanoparticle size, morphology, surface chemistry, and functional performance. Consequently, the lack of standardized synthesis protocols limits inter-study comparability and hinders industrial and regulatory acceptance.

### 7.1. Scale-Up and Standardization Issues

Translating phyto-mediated nanomaterial synthesis from laboratory-scale production to industrial implementation remains a major challenge. Although small-scale synthesis is generally simple and cost-effective, large-scale manufacturing requires precise control over reaction parameters, consistent raw material supply, and reproducible product quality. Variations in plant biomass availability, extraction efficiency, and processing conditions can significantly complicate scale-up efforts. Consequently, the development of standardized synthesis protocols, scalable reactor systems, and quality-control strategies remains an important research priority [166]. A major obstacle during scale-up is the batch-to-batch inconsistency associated with biological precursors. Unlike highly purified chemical reagents, plant extracts naturally vary in the concentration and composition of reducing and stabilizing agents such as polyphenols and flavonoids depending on plant age, geographical origin, soil conditions, and harvest season. To address these limitations, recent studies have increasingly explored continuous-flow synthesis systems, which provide improved heat and mass transfer compared with conventional batch reactors, thereby enhancing nanoparticle uniformity and process reproducibility at industrially relevant scales.

### 7.2. Variability in Plant Extract Composition

The chemical composition of plant extracts varies considerably depending on plant species, tissue type, growth conditions, seasonal variations, extraction procedures, and storage conditions. Such variability directly influences nanoparticle reduction, stabilization, surface functionalization, and overall physicochemical behavior, often leading to inconsistent material performance. In addition, the limited mechanistic understanding of the individual roles of specific phytochemicals further complicates predictive nanomaterial design and optimization. Therefore, advanced analytical characterization, metabolomic profiling, and mechanistic investigations are required to establish clearer relationships between extract composition and nanomaterial functionality. Future research should also focus on phytochemical fingerprinting, standardized extract libraries, and AI-assisted optimization approaches to improve synthesis predictability and reproducibility.

### 7.3. Regulatory and Policy Challenges

Regulatory frameworks governing nanomaterials are still evolving and often do not explicitly address phyto-mediated or biologically synthesized systems. Existing regulations are commonly adapted from conventional chemical or engineered nanomaterial guidelines and may therefore inadequately capture the unique surface chemistry, environmental behavior, and transformation pathways associated with plant-derived nanomaterials [167]. The absence of clear regulatory pathways, standardized safety assessment procedures, and internationally harmonized policies remains a significant barrier to commercialization and environmental deployment. Furthermore, inconsistencies in characterization methods, toxicity evaluation protocols, and reporting standards complicate cross-study comparisons and regulatory approval processes. Coordinated collaboration among researchers, regulatory agencies, industry stakeholders, and policymakers will therefore be essential for establishing effective governance frameworks that support the safe and sustainable implementation of phyto-mediated nanotechnologies [168].

## 8. Future Perspectives and Direction

Phyto-mediated nanomaterials represent a convergence of innovation, advanced nanotechnology, and environmental sustainability for next-generation remediation technologies. Future progress in this field will depend not only on advances in material design and mechanistic understanding (Figure 5), but also on the integration of hybrid systems, standardized production strategies, and robust sustainability assessment frameworks. From a scientific and technological perspective, future research should focus on improving control over nanoparticle synthesis through a deeper understanding of the interactions between phytochemicals and nanomaterial surfaces. Identifying the specific phytochemicals responsible for reduction, stabilization, and surface functionalization will enable the development of more predictable and tunable nanomaterials with tailored environmental performance. The integration of advanced characterization techniques, computational modeling, machine learning, and data-driven optimization approaches may further accelerate the rational design of PMNs for targeted remediation applications. Another important direction in green nanochemistry is the development of hybrid nanomaterial systems in which phyto-mediated nanoparticles are integrated with complementary materials such as carbon-based supports, polymeric matrices, biochar, or doped semiconductor frameworks. These hybrid architectures can improve catalytic efficiency, stability, selectivity, and reusability while enabling multifunctional “adsorb–catalyze–sense” platforms capable of treating complex environmental matrices. In particular, coupling biogenic nanomaterials with visible-light-responsive semiconductors or conductive carbonaceous support offers promising opportunities for the remediation of persistent pollutants under realistic environmental conditions.

Sustainability considerations must remain central to the large-scale deployment of PMNs. Future studies should therefore incorporate comprehensive life-cycle assessment (LCA) and techno-economic assessment (TEA) approaches to evaluate environmental impacts, process efficiency, scalability, and economic feasibility across the entire value chain. Such analyses will help identify critical trade-offs and guide the transition from laboratory-scale synthesis to industrial implementation. Furthermore, the development of closed-loop systems for nanomaterial recovery, regeneration, and reuse, together with integration into renewable-energy-driven and nature-based treatment systems, may further strengthen the sustainability profile of phyto-mediated nanotechnologies and support circular economy strategies [169]. Despite the promising advances achieved thus far, important uncertainties remain regarding long-term environmental behavior, large-scale reproducibility, environmental fate, and regulatory acceptance. Therefore, future progress will also depend on the establishment of standardized testing protocols, harmonized safety assessment frameworks, and adaptive regulatory guidelines specifically designed for green and biologically derived nanomaterials. Collaborative engagement among researchers, policymakers, regulatory agencies, and industrial stakeholders will be essential to ensure responsible innovation, environmental protection, and safe commercialization of phyto-mediated nanotechnologies.

## 9. Conclusions and Outlook

PMNs synthesized using plant extracts represent a promising green and sustainable alternative to conventional nanomaterial synthesis methods. Plant-derived phytochemicals function as natural reducing, stabilizing, and capping agents, imparting enhanced biocompatibility, surface functionality, and improved pollutant selectivity across water, soil, and air remediation systems. Compared with conventional nanomaterials, phyto-mediated systems offer significant advantages in terms of reduced chemical toxicity, lower energy requirements, and alignment with green chemistry and circular economy principles. However, their transition from laboratory-scale synthesis to practical environmental technologies is constrained by three major bottlenecks: extract batch variability, insufficient ecotoxicity assessment, and limited industrial standardization.

First, batch-to-batch variations in plant extract composition can alter nanoparticle size, surface chemistry, and remediation performance, requiring standardized extraction procedures and phytochemical characterization. Second, comprehensive ecotoxicity assessment is needed to determine the long-term fate, transformation, bioaccumulation, and ecological effects of PMNs and their degradation products under realistic environmental conditions. Third, industrial translation requires standardized synthesis and quality-control protocols, reproducible scale-up procedures, and integration of techno-economic and life-cycle assessments. Addressing these three priorities will be essential for establishing reliable performance, environmental safety, and regulatory acceptance of PMNs.

Overall, PMNs have considerable potential for sustainable water treatment, environmental remediation, carbon management, and climate-related applications. Future progress should therefore prioritize reproducibility, environmental safety, and scalable standardization rather than laboratory-scale performance alone, enabling the responsible transition of PMNs toward practical environmental deployment.

## Figures and Tables

**Table 1:** Comparative characteristics, application-based selection, and environmental implications of conventional nanomaterials (CNMs) and phyto-mediated nanomaterials (PMNs).

Criteria	Conventional Nanomaterials	Phyto-Mediated Nanomaterials	Application-Based Selection	Environmental Implication of Phyto-Mediated Nanomaterials
Synthesis method	Chemical reduction using synthetic reducing agent (e.g., NaBH_4_), sol-gel, hydrothermal, physical vapor deposition	Green synthesis using plant phytochemicals as reducing and stabilizing agent	Select conventional methods when precise control and reproducibility are critical; use phyto-mediated for eco-friendly synthesis	Reduced chemical toxicity and lower environmental footprint
Reproducibility	High reproducibility	Batch-to-batch variability	Conventional systems better for industrial standardization	Variability in plant extracts may affect consistency and require improved synthesis standardization.
Surface Chemistry	Requires post-synthesis functionalization	Naturally capped with phytochemicals (polyphenols, phenolic proteins)	Phyto-mediated advantageous for adsorption, biocompatibility, and stability	Enhance adsorption affinity and redox activity
Toxicity	Use toxic reagents/surfactants causing risk of secondary pollution and long-term environmental impact.	Reduced toxicity and improved biocompatibility	Phyto-mediated ideal for environmental and biomedical applications	Potentially lower residual chemical toxicity; however, long-term ecotoxicity and environmental fate require further assessment.
Energy & Resource Demand	Energy-intensive (high temperature/pressure). Top-down method	Often synthesized under comparatively mild and lower-energy conditions	Phyto-mediated preferred for sustainable and low-cost production, especially in resource-limited settings	Preferentially facilitate circular economy and waste upcycling
Cost	Higher due to energy and chemicals	Generally lower-cost (biomass-based)	Phyto-mediated preferred for low-cost water treatment	Lower long-term environmental impact.
Scalability	Established industrial scalability	Scaling challenges (biomass variability, extraction)	Conventional for large-scale uniform production; phyto for decentralized systems	Requires standardization for large-scale applications.
Mechanism	Requires engineered doping/modification	Natural functional groups enhance adsorption & redox activity	Material selection should consider the target pollutant and required removal mechanism; PMNs may provide additional phytochemical-mediated surface interactions	Bio-derived surface functionalities may influence contaminant interactions and environmental behavior
Particle Size & Morphology Control	Highly controlled and tunable	Less precise; variability due to plant extract composition	Conventionally preferred for electronics, catalysis requiring uniformity	Improved synthesis control is required to achieve consistent particle properties and environmental performance.
Stability (pH/Redox)	May require stabilizers; prone to aggregation	Biogenic coatings improve colloidal stability	Phyto-mediated suitable for complex environmental matrices	Stability depends on environmental conditions, including pH, ionic strength, natural organic matter, and degradation of the biogenic corona.

Note: The relative advantages of CNMs and PMNs are application-dependent; material selection should therefore consider performance requirements, reproducibility, environmental conditions, scalability, and long-term safety.

**Table 2:** Representative studies reporting on the application of phyto-mediated nanomaterials in environmental remediation.

Plant	Phyto-Mediated Nanomaterials	Application	Medium	Results	Reference
*Ficus exasperata* leaves.	Mn-Mg oxide NPs	Remediation of heavy-metal-contaminated soil affected by spent engine oil	Soil	Mn-Mg NPs showed potency in immobilizing Ni, Cu, Zn, Fe, and Cr in contaminated soils	[56]
*Hydrocotyle umbellata* leaves	Mg-doped FeNPs	Adsorption of Ni^2+^ ions	Water	Increased adsorption capacity of Ni^2+^ ions (84.74 mg/g) over commercial nanoparticles	[57]
*Trigonella hamosa* leaves	AgNPs	Elimination of organic pollutants	Water	Photodegradation of methylene blue dye (94.49%) and paracetamol (92%) from polluted water	[58]
*Phaseolus vulgaris*	AgNPs	Photocatalytic removal of biological and non-biological pollutants	Water	Demonstrated photocatalytic, catalytic, and antimicrobial activity for environmental remediation	[59]
*Azadirachta indica* (neem) and *Mentha longifolia* (mint) leaves	Nano-zero valent iron	Remediation of Pb- and Ni-contaminated soil	Soil	Synthesized neem and mint nZVI particles had the potential to remove Pb and Ni from contaminated soil	[60]
*Mentha piperita leaf extract*	nZVI (nano zero-valent iron)	Canal water remediation	Water	Removed phosphate (85.0%), ammonia (99.5%), nitrate (86.3%), chloride (83.0%) and Pb (79.3%) within 24 h	[61]
*Macrotyloma uniflorum (horse gram) seed*	CeO_2_ and selenium doped CeO_2_	climate-smart agriculture	Agricultural system	Enhanced seedling vigor	[62]
*Murraya koeignii*	lanthanum ferrite (BC/LaF) nanohybrid structure	Removal of antibiotics from water	Water	Removal of ciprofloxacin from contaminated water	[63]

Note: PMN performance varies with plant source, nanomaterial composition, target pollutant, treatment medium, and experimental conditions; therefore, direct quantitative comparison among the reported studies should be interpreted with caution.

## Data Availability

No new data were created or analyzed in this study. Data sharing is not applicable to this article.
